# Development of a Widely Accessible, Advanced Large-Scale Microfluidic Airway-on-Chip

**DOI:** 10.3390/bioengineering12020182

**Published:** 2025-02-13

**Authors:** Brady Rae, Gwenda F. Vasse, Jalal Mosayebi, Maarten van den Berge, Simon D. Pouwels, Irene H. Heijink

**Affiliations:** 1 Department of Pathology & Medical Biology, University Medical Center Groningen, University of Groningen, 9713 GZ Groningen, The Netherlandsm.van.den.berge@umcg.nl (M.v.d.B.); s.d.pouwels@umcg.nl (S.D.P.); h.i.heijink@umcg.nl (I.H.H.); 2 University Medical Center Groningen, GRIAC Research Institute, University of Groningen, 9713 GZ Groningen, The Netherlands; 3 Department of Respiratory Medicine and NUTRIM School of Nutrition and Translational Research in Metabolism, Maastricht University, 6211 LK Maastricht, The Netherlands; j.mosayebi@maastrichtuniversity.nl; 4 Department of Pulmonary Diseases, University Medical Center Groningen, University of Groningen, 9713 GZ Groningen, The Netherlands

**Keywords:** microfluidics, airway-on-chip, epithelial cells, PDMS, air–liquid interface

## Abstract

On-chip microfluidics are advanced in vitro models that simulate lung tissue’s native 3D environment more closely than static 2D models to investigate the complex lung architecture and multifactorial processes that lead to pulmonary disease. Current microfluidic systems can be restrictive in the quantities of biological sample that can be retrieved from a single micro-channel, such as RNA, protein, and supernatant. Here, we describe a newly developed large-scale airway-on-chip model that employs a surface area for a cell culture wider than that in currently available systems. This enables the collection of samples comparable in volume to traditional cell culture systems, making the device applicable to any workflow utilizing these static systems (RNA isolation, ELISA, etc.). With our construction method, this larger culture area allows for easier handling, the potential for a wide range of exposures, as well as the collection of low-quantity samples (e.g., volatiles or mitochondrial RNA). The model consists of two large polydimethylsiloxane (PDMS) cell culture chambers under an independent flow of medium or air, separated by a semi-permeable polyethylene (PET) cell culture membrane (23 μm thick, 0.4 μm pore size). Each chamber carries a 5 × 18 mm, 90 mm^2^ (92 mm^2^ with tapered chamber inlets) surface area that can contain up to 1–2 × 10^4^ adherent structural lung cells and can be utilized for close contact co-culture studies of different lung cell types, including airway epithelial cells, fibroblasts, smooth muscle cells, and endothelial cells. The parallel bi-chambered design of the chip allows for epithelial cells to be cultured at the air–liquid interface (ALI) and differentiation into a dense, multi-layered, pseudostratified epithelium under biological flow rates. This millifluidic airway-on-chip advances the field by providing a readily reproducible, easily adjustable, and cost-effective large-scale fluidic 3D airway cell culture platform.

## 1. Introduction

Recently, on-chip technologies have been increasingly utilized to study the development and progression of respiratory diseases in vitro in order to represent the complexity of the human lung architecture more closely. These in vitro systems advance on static models by facilitating the study of specific interactions between different cell types, 3D mechanical forces, environmental factors, and the underlying extracellular matrix (ECM) [1]. Further, by recreating the 3D tissue environment, responses of the resident cells reflect the in vivo situation better than standard 2D culture models do. In vivo, paracrine signaling molecules are in constant motion, conveyed by the interstitial flows produced by diffusion from the surrounding vasculature. These flows facilitate the development of local concentration gradients and directional cell–cell communication, strongly influencing cell behavior. Moreover, at the surface of the respiratory tract, epithelial cells are exposed to continuous flows of air, stimulating their surface mechanoreceptors. This airflow-induced shear stress promotes cell differentiation and directional mucociliary organization [2]. Recreating these flows in vitro takes us a step further towards bridging the gap between the understanding of biological responses in the lab and their relevance for the clinic. Furthermore, it facilitates the study of therapeutic interventions on interactions not present in static cell cultures and reduces the necessity of the involvement of animal models. Providing accessible dynamic platforms available to many lab environments is a key step in making these advances the standard.

The core function of the airway epithelium is to act as a barrier between the internal and external environments, protecting from environmental insults such as pathogens. Maintaining a healthy and responsive semi-permeable size-selective barrier requires active communication between the cells that populate the airway wall [3]. Airway epithelial cells maintain a durable physical barrier through the formation of tight and adherens junctions between adjacent cells via interactions between transmembrane proteins such as Zonula occludens 1 (ZO-1) and E-cadherin. This is required for their polarization and differentiation into a pseudostratified, mucus producing epithelial layer [4].

To investigate the airway barrier in a pseudostratified epithelial layer, epithelial cells can be cultured at ALI. Air exposure promotes barrier formation through notch inhibition, leading to cell differentiation into secretory club cells, goblet cells, and ciliated cells, among other cell types, recapitulating the in vivo composition of the tissue [5]. The two chambers in conventional ALI models are separated by a porous membrane that facilitates the introduction of other cell types to investigate the effects of cell–cell communication, such as fibroblasts’ influence on epithelial mucus secretion [6,7]. Through the application of advanced in vitro models that include more elements of the native 3D environment, more complex interactions that contribute to respiratory disease can be investigated. In microfluidic models, this includes the addition of directional flow, the resultant mechanical stimulation, and planar polarization of the different cell types in the model [8,9].

Many configurations of airway-on-chip have been developed. This device is most comparable to the vertically stacked configuration models, with the most well-known of this type being the Wyss Institute devices such as the PDMS model from Emulate. The emulate model is smaller and capable of cyclical membrane stretch with its own dedicated pump and flow management systems [10,11]. Our device has an open-access design, the membranes can be easily removed and replaced, and potentially any flow management system can be attached to it. This exchanges the plug-and-play elements of commercial systems such as the emulate platform for a wide range of applications and integration into different experimental setups. Additionally, while commercial systems come with a financial investment, open access models like ours are significantly more cost effective in exchange for an investment of time that facilitates more replicates and exploratory research. While our device is focused on exposures and wide accessibility of different samples, other models are designed to investigate the physiological element of the respiratory tract by specializing in applying different biological shear stresses on cells during differentiation [12] or how pharmacological/infectious agents travel through the different compartments of the respiratory tract [13]. Other airway-on-chip devices aim to reproduce structural elements of the airway such as the tubular nature of the airway or alveoli [14] or the mechanical influences of introducing fibrotic-like properties and materials to stretchable membranes [15]. Use of our novel dynamic models will make possible more insightful dynamic investigations into epithelial barrier dysfunction and dysregulation of differentiation/regeneration upon injury, an important pathological feature of respiratory diseases like chronic obstructive pulmonary disease (COPD) [4,7].

Furthermore, the advancement of airway-on-chip technologies has facilitated the introduction of aerosolized drugs and whole particulate such as cigarette smoke into cultures at ALI [16,17,18]. Previously, the responses of human bronchial epithelial cells to environmental factors such as cigarette smoke, environmental pollutants, and viruses have been investigated in submerged 2D monoculture systems in well plates [6,16]. These studies have been furthered into Transwell systems where bronchial epithelial cells are differentiated statically in 3D ALI cultures [7].

Although current airway-on-chip models are a huge step forwards, many currently available microfluidic systems are micro-scale devices with highly specialized construction methods, often with a static top chamber to supply a fresh medium for the culture [16,17,18,19]. While a variety of truly dynamic systems are available, requirements for specialized construction facilities means that the focus remains on commercial platforms which often overcome these restrictions with costs that also limit accessibility [20]. Here, we present a newly developed, large millifluidic-scale airway-on-chip consisting of two PDMS chambers divided by a PET cell culture membrane (23 μm thick, 0.4 μm pore size, 11.6%/1.6 × 10^6^ cm^−2^). Unlike other devices (≤1 mm), this airway-on-chip contains a wide (5 mm) directional apical chamber providing a larger number of cells than is common in current airway-on-chip devices. The construction methodology that we have developed produces a flexible and water-tight seal with robust membrane placement by slow-curing the PDMS sealant under a vacuum. With the accessibility and size of this platform, we provide a means to move into a 3D culture while employing traditional, material-dependent lab techniques. To assess the device’s function as a dynamic ALI platform, human airway epithelial cells were cultured, exposed to dynamic airflow, and shown to differentiate into a pseudostratified epithelium under these conditions within the device. In addition, to mimic the in vivo situation more closely, with extensive cell–cell communication between the epithelia and underlying mesenchyme, we co-cultured the airway epithelial cells with airway fibroblasts on the other side of the membrane. The developed device provides 92 mm^2^ of dynamic cell culture area with air exposure in a readily adaptable and reproducible airway-on-chip platform. As such, this device facilitates the sampling of large volumes of cells and media to study multiple read-outs.

## 2. Materials and Methods

### 2.1. Microfluidic Device Preparation

Separate molds were designed for each half of the chip using CAD software SolidWorks Premium 2021 and the G-code for milling was produced in SolidWorks Cam (SOLIDWORKS Corp., Waltham, MA, USA). Files in Standard Tessellation Language (.STL) format are available in the online supplement. Subsequently, the molds were milled from solid Polymethyl methacrylate (PMMA; Colltec, Groningen, NL, USA) using a Minitech CNC Mini-Mill/GX (Minitech Machinery Corp., Nocrcross, GA, USA) with 1.5 mm mill bits (N.SA.1,5.30°.Z2.HA.K7,5/43 DPA72S; Ceratizit group, Roozendaal, NL, USA).

To prepare the chip halves from these molds, a PDMS Elastomer base was mixed with a curing agent (Sylgard 184 Silicone Elastomer base and curing agent, Dow Corning, Midland, MI, USA) at a 10:1 weight ratio. The solution was thoroughly mixed, degassed for 30 min in a vacuum chamber, and injected into the molds with a syringe. The molds were then carefully transferred to a dry oven (65 °C, 2 h) for curing. PDMS chip halves were carefully removed from the molds using a scalpel to remove any flashing, and four inlets were punched into the top half. Debris was removed from the chip halves with Scotch tape™ (3M, Saint Paul, MN, USA) before UV sterilization (365 nm 9-watt generic UV bulbs, 20 min). Chip halves were then stored at 4 °C under tape in sealed Petri dishes for up to a month before the final construction.

**Figure 1:**
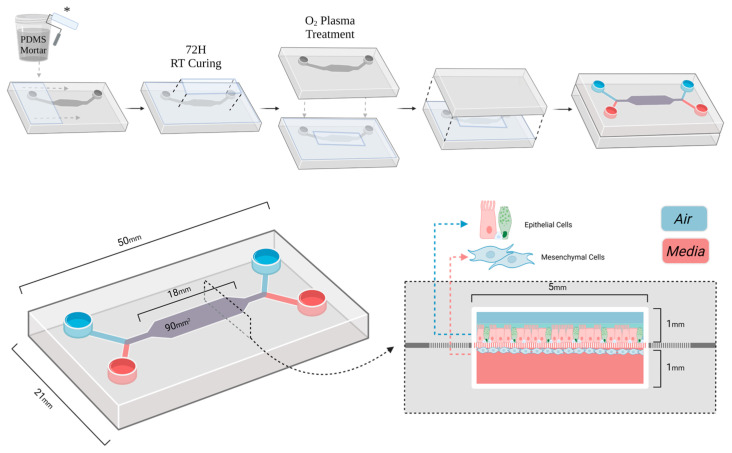
Chip construction procedure. Detailed step-by-step visual guide to sealing a chip via the stamp method. Prepared and sterilized PDMS chip halves were rolled with a PDMS Mortar (5:7 PDMS–toluene) with a chemical-resistant rubber roller (*). The pre-cut and coated membrane was placed on the wet PDMS, mortar was applied to the top, and it was placed under a vacuum for 72 h to cure at RT. Both chip halves were treated in a plasma cleaning oven (320 mBarr 30 s) and sealed together with the application of a little manual force. The device was then prepared for cell seeding, an example of this can be seen with air-exposed epithelial cells and mesenchymal cells in co-culture. Created with BioRender.com.

Cut membranes were coated in minimum essential Eagle’s medium (EMEM; Lonza, Basel, Switzerland), containing 3 mg/mL of PureCol (Collagen; Advanced BioMatrix, Carlsbad, CA, USA), 1 mg/mL of fibronectin bovine plasma (Sigma-Aldrich, Burlington, MA, USA), and 10 mg/mL of bovine serum albumin (BSA; Sigma-Aldrich), overnight at 37 °C before being washed twice with EMEM and stored at 4 °C for up to a month before the final construction. Finally, top halves were cleared of debris with Scotch tape^TM^ before PDMS mortar (7:5 PDMS:toluene; Merck Millipore, Darmstadt, Germany) was applied to the inner surface with a roller [13]. Toluene was used to thin the liquid PDMS as they mix well and it has low viscosity and high volatility. The PET in the membrane is known to display resistance to attack from toluene, which can readily be removed from the device under a vacuum. Pre-coated membranes were carefully placed on the inner surface and mortar was then applied on top of the edges of the membrane before curing. Wet chip halves with membranes were placed in a chamber under a vacuum for 72 h of curing at room temperature. Membrane bound chips were sealed via O_2_ Plasma treatment (320 mBarr, 30 s; Harrick Plasma, Ithaca, NY, USA; Figure 1). Completed device inlets were covered with tape before being stored in Petri dishes at 4 °C until cell seeding (Figure 1).

### 2.2. Platform Validation

To validate the function of our device as a dynamic ALI platform and a suitable replacement for traditional culture platforms, the following series of quantifications and comparisons were conducted in the differentiating human lung adenocarcinoma Calu-3 cell line (ATCC, Manassas, VA, USA), which was able to differentiate into mucus-producing cells upon ALI exposure. By showing these comparisons in a standardized ALI cell line, we provide tangible evidence of the model’s function in relation to other platforms shown here and in the literature.

#### 2.2.1. Cell Culture

The human lung adenocarcinoma epithelial Calu-3 cell line was cultured in a medium consisting of 1:1 DMEM (Dulbecco’s modified Eagle’s medium, Lonza, Switzerland 12-707F) and Ham’s F12 (Gibco, San Diego, CA, USA, 11765-054) supplemented with 10% fetal bovine serum (FBS; Serana, Germany) + 1% non-essential amino acids (Gibco, Stockton, CA, USA) + 10,000 units/mL penicillin/streptomycin (Gibco) at 37 °C and 5% CO_2_.

The cells were grown in 25 cm^2^ culture flasks and passaged with trypsin/EDTA once they were visually confirmed to be at ~90% confluency using light microscopy.

#### 2.2.2. Cell Seeding in the Millifluidic Airway-on-Chip Device

**Figure 2:**
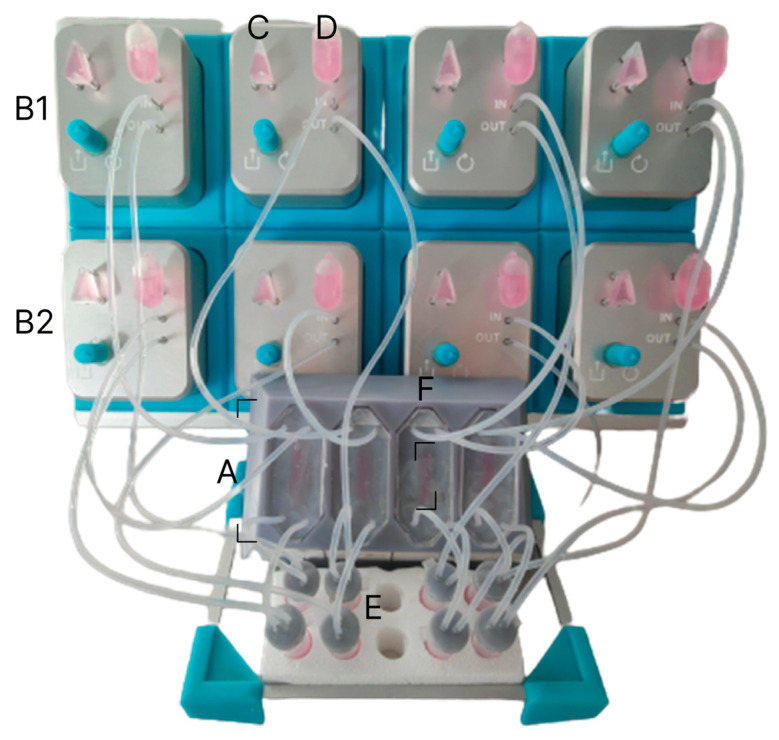
Complete experimental setup of the large scale airway-on-chip. Four chips attached to the Porcupine peristaltic pump (A). Each of the chambers in the chips was attached to a separate pump (B1/2), on the left of each pump the triangular bubble trap (C) can be seen, and on the right is the 1 mL media reservoir (D). Before the medium flowed into the chip, it was dropped into an Eppendorf that reduced flow variation (E). Cell culture areas of 92 mm^2^ can be seen being cultured and submerged in the center of each device (F).

Calu-3 cells were seeded into the chips at a density of 6.5 × 10^4^ cells in 100 μL of culture medium and incubated overnight statically at 37 °C and 5% CO_2_ for cell attachment. Subsequently, the chips were connected to the a Porcupine peristaltic pump (IMcoMET, Rotterdam, NL, USA; Figure 2).

#### 2.2.3. In-Chip Cell Count Quantification

Calu-3 cells were seeded in the chips, grown to confluence, and trypsinized as described above. The cells were seeded in 24 well plates, 6.5 mm Transwell inserts, and chips in parallel with 1 × 10^5^ cells, 4 × 10^4^, and 6.5 × 10^4^ cells, respectively. Upon reaching >90% confluency, the cells were exposed to air, unless in wells, and maintained for 10 days. Full medium changes were performed every three days. The cells were trypsinized as described above, and cell numbers for the wells, inserts, and chips were counted using a Bürker-Türk counting chamber according to the standard protocol [21,22]. Cells obtained from the 4 replicates were each counted twice and averaged (Table 1).

#### 2.2.4. Trypan Blue and AlamarBlue Cell Viability Assays in the Chip Device

The chips were prepared and sealed as above in the presence or absence of toluene in a PDMS mortar (7:5 PDMS:toluene). Calu-3 cells were then seeded into the devices and grown to confluency as described above. After 24 h, the supernatant was collected, and an AlamarBlue^®^ cell viability assay was performed according to manufacturer’s instructions (Thermo Fischer Inc., Stockton, CA, USA). Fluorescence was recorded on a VICTOR3™ Multilabel Plate Reader (PerkinElmer Inc., Waltham, MA, USA). Cells on the membranes were then stained with Trypan blue (1:1 HBSS, Hanks’ balanced salt solution, Gibco, San Diego, CA, USA) for 60 s, and the dead cells were counted.

#### 2.2.5. Assessment of Shear Stress Applied to the Cell Layer in the Chip Device

To understand how media flow through the device exerts shear stress on the cell layer, flow rate was converted to shear stress through the formula:

(1) $$\tau =\frac{6Q\mu }{{WH}^{2}}$$

Here, τ is shear stress (dyn/cm^2^), Q is the flow rate (μL/min), μ is viscosity (mPa·s), W is width, and H the height (mm) of the channel. The formula itself is a derivative of the laws determining behaviors of Newtonian fluids in rectangular chambers [19]. Viscosities of the DMEM with 10% FCS and local air from an incubator at 100% humidity were considered respectively as 0.958 and 0.018 cP. The shear stress produced by media flow at a rate of 150 μL/hour within the device was calculated to be 5 × 10^−4^ dyn/cm^−2^ [23,24].

#### 2.2.6. Fluid Dynamic Analysis Within the 3D Geometry of the Device

Fluid dynamic analysis was performed using COMSOL Multiphysics (COMSOL Inc., version 5.5, 2020, Stockholm, Sweden). The designed microfluidic geometries were imported from SolidWorks software (SolidWorks, Version SP0, 2019, Dassault Systems, Waltham, MA, USA) using the LiveLink function of COMSOL Multiphysics. In microfluidic devices with small microchannel geometric length scales, the movement of the fluid is governed by the continuity Equation (2) and the Navier–Stokes Equation (3) [25]. A physics-controlled mesh was used with the element size set to extra fine.

(2) $$\nabla \cdot \bar{U}=0$$

(3) ρ(∂U¯^∂t^+U¯·∇U¯)=−∇p+ρg¯+∇·[τ¯]

In these equations, ρ is the density of the fluid (kg/m^3^), U = (u, v, w) is the flow velocity vector (m/s), *p* is pressure (Pa), ∇ is divergence, and [τ¯] is the shear stress tensor in Pa.

The computational fluid dynamic (CFD) simulation was implemented to solve the incompressible Navier–Stokes Equation (3) and the continuity Equation (2) to evaluate the velocity fields, the shear stresses, and the pressure inside the different microchannel geometries.

The type of physics selected to solve the simulations was single-phase laminarflow, which is suitable for small-geometrical-length-scale microfluidic devices. The steady-state condition was selected. Subsequently, a no-slip boundary condition was applied to the walls. Flow rate 2.5 µL/min was used as inlet conditions for the four different microchannel geometries. The pressure at the outlets was set at P = 0 Pa, which refers to the inlets (no backpressure). Additionally, water was considered as a reference fluid to run the simulations as it is a Newtonian fluid, meaning that its shear stress (*τ*) is proportional to the shear rate ($\dot{y}$), and *μ* is the fluid viscosity according to Equation (4) [26].

(4) $$\tau =\mu \dot{y}$$

The Reynolds number as the main controlling parameter in fluid flow within the microfluidic channels is less than 1; as a consequence, the fluid within the microchannels is laminar. In addition, for the designed microchannel geometries, the fluid flow is laminar throughout the microchannel due to the low flow rate. Thus, this chip design does not induce any air or fluid turbulences that may either disrupt or damage the cells [26].

#### 2.2.7. Velocity Profiles Characteristics

For a rectangular cross-section microchannel of width w and height h, the velocity profile can be determined by solving the Navier–Stokes Equation (2) using the Fourier series [27]:

(5) $$v(x,z)=\frac{48Q}{\left(z^{3}\cdot h\cdot w\right)}\cdot \frac{\sum \frac{\infty }{l}=odd\frac{1}{l^{3}}[1-\frac{\cos h\left(\pi \frac{x}{h}\right)}{\cos h\left(\pi \frac{w}{2h}\right)}sin(iz\frac{z}{h})]}{1-\sum \frac{\infty }{l}=odd\frac{1}{l^{5}}\cdot \frac{192}{\pi^{3}}\cdot \frac{h}{w}\tan h(ix\frac{w}{2h})}$$

From Equation (5) [28,29], the velocity profile has a different distribution along the x- and z-directions. The velocity profile is parabolic, whereas along the larger dimension, the velocity shows a plateau in the center and then changes near the wall [30].

As shown by Yun et al. [30], as the ratio between the height (h) and the width (w) of the microchannel decreases, the velocity profile tends to flatten in the center of the microchannel, showing a plateau region. Secondly, it is important to highlight the role of wall shear stress, which is a mechanical stimulus generated by the friction of the fluid against both the channel walls and the apical cell membranes.

### 2.3. Computational Meshing for CFD Simulations

A computational mesh study was conducted on the four designed geometries using COMSOL Multiphysics. For this purpose, hybrid meshes with tetrahedral, pyramidal, and prismatic element types were generated. For each designed geometry, color mesh plots were created to understand where low-quality elements were positioned. A quality mesh of 1 indicates an optimal element quality, whereas O represents a degenerated element. In general, elements with a quality below 0.1 are considered of poor quality. The same mesh generation process was implemented for each designed geometry.

#### 2.3.1. Cell Differentiation Under Airflow in the Millifluidic Device

Upon reaching confluency, Calu-3 cells were either exposed to air or maintained submerged under a continuous flow for 10 days at a constant rate of 150 μL/h, to provide the apical mechanical airflow stimulation established above and continuous supply of fresh media basolaterally at a rate of 150 μL/h. Circulating media was replaced with a full 1 mL reservoir of medium every 3 days. Cell-free supernatants and apical washes were collected at day 10 (Calu-3) and stored at −20 °C for up to two weeks before ELISAs were performed. For the secretion of mucus into the apical compartment, the chips were incubated statically in 100 μL of respective media for 1 h before apical wash was applied for 5 min and immediately stored at −20 °C for up to two weeks before performing the ELISA. The cells were washed with phosphate-buffered saline (PBS), fixed in 4% paraformaldehyde (PFA) for 20 min, and whole chips were stored in PBS at 4 °C before staining.

#### 2.3.2. In-Chip Total Protein Quantification from Supernatant

Calu-3 cells grown to confluency in 24 well plates, Transwell systems, or the airway-on-chip device were serum-deprived for 24 h before the supernatants were collected for quantification of total protein content. Total protein yield was determined using a Pierce BCA protein assay kit (Thermo Fisher Scientific Inc., Waltham, MA, USA), according to the manufacturer’s instructions. Absorbance at 550 nm was recorded with a CLARIOstarPlus (BGM Labtech, Ortenberg, Germany), and absolute concentrations were calculated from the standard curve.

#### 2.3.3. In-Chip Measurement of Released IL-8 from Supernatant

Released IL-8 was measured in cell-free supernatants with a Human IL-8/CXCL8 DuoSet ELISA kit (Catalog #: DY208. R&D Systems, Minneapolis, MN, USA) according to the manufacturer’s instructions.

#### 2.3.4. In-Chip RNA Quantification

Calu-3 cells grown to confluency in 24-well plates, Transwell systems, or the airway-on-chip device were treated with HBSS (2×, 5 min), trypsinized from the apical chamber, and centrifuged at 590× *g* for 5 min. The pellets were lysed into 100 μL of Trizol, which was subsequently stored at −20 °C until RNA isolation. The samples were defrosted and allowed to incubate at room temperature for 5 min for nucleoprotein dissociation.

The sample was incubated further for 3 min with 20 μL of chloroform and centrifuged at 12,000× *g* 4 °C. The apical, aqueous phase was removed and incubated for 10 min in 50 μL of isopropanol before centrifuging at 12,000× *g* 4 °C. The supernatant was removed, and the RNA pellet was resuspended in 100 μL of 75% ethanol, vortexed, and centrifuged for 5 min at 7500× *g* 4 °C. Next, the supernatant was discarded, while the pellet was air-dried and subsequently resuspended in 20 μL of RNAse-free water. RNA quantity was assessed using a NanoPhotometer^®^ N60-MOBILE UV/Visible Spectrophotometer (VWR, Radnor, PA, USA).

#### 2.3.5. Quantification of MUC5AC by ELISA

Cell-free supernatants were prepared by centrifuging for 20 min at 1000× *g*. Subsequently, the supernatants were diluted 1:1 with PBS, and MUC5AC ELISA was performed according to manufacturer’s instructions (Cloud-Clone Corp., Houston, TX, USA). Absorbance at 450 nm was recorded with a CLARIOstarPlus (BGM Labtech).

#### 2.3.6. Embedding of the Cells Cultured Within the Chips

The cells were washed with PBS, fixed in 4% PFA for 20 min, and stored in PBS at 4 °C before staining. The membranes were carefully removed and cut into ~3 × 10 mm segments and embedded in paraffin. Paraffin blocks were cut 4 μm thick and mounted on StarFrost^®^ glass slides (Knittel glass, Braunschweig, Germany). The samples were deparaffinized and rehydrated immediately before staining.

#### 2.3.7. Alcian Blue Staining for Detection of Mucins

Membrane segments were then immersed in an Alcian Blue solution (Abcam, Cambridge, UK) for 30 min and washed in running tap water for 2 min before immersing in demi-water. The samples were counterstained with a Nuclear Fast Red solution (Abcam) for 5 min and washed in running tap water for 1 min. The slides were then dehydrated, cleared with xylene, and mounted with Permount ™ (Thermo Fisher Scientific Inc.). Images were captured on a Leica DM IL LED Inverted Laboratory Microscope with a Leica MC120 HD 2.5 Megapixel HD Microscope Camera (Leica Microsystems, Wetzlar, Germany).

#### 2.3.8. Immunofluorescent Staining

The slides were deparaffinized, rehydrated, and antigen retrieval was conducted in a Tris/EDTA buffer (10/1 mM) by heating for 15 min in the microwave before cooling to room temperature. Membrane segments were washed three times with PBS and incubated at room temperature for 1 h in 5% BSA for blocking, washed with PBS and incubated for 1 h with the mouse anti-MUC5AC antibody (1:200, AbCam, Cambridge, UK), anti-E-cadherin antibody (1:500, Santa Cruz Biotechnology, Santa Cruz, CA, USA), or mouse anti-ZO-1 antibody (1:200, Thermo Fisher Scientific Inc.) in PBS containing 1% BSA. After washing with PBS, the cells were incubated for 1 h with AlexaFluor 555 anti-mouse conjugated secondary antibody (1:200, Abcam) in PBS containing 1% BSA. Finally, the samples were incubated for 10 min in DAPI in PBS (1 µg/mL). The cells were washed in PBS before fixing under CitiFluor (VWR). Images were taken with an EVOS™ FL Digital Inverted fluorescence Microscope using identical settings, and the images were pseudo-colored for the different targets (Thermo Fisher Scientific Inc.).

### 2.4. A Dynamic Primary Respiratory Cell Co-Culture ALI Platform

To show the full intended function of the platform for primary airway cells, long-term culture, and co-culture under a dynamic biological flow, primary cells isolated from human donor tracheobronchial tissue were cultured in the chip device as described below.

#### 2.4.1. Primary Cell Culture

Human airway epithelial cells (AECs) were isolated by enzymatic treatment of leftover tracheobronchial tissue from 3 healthy lung donors, from whom no further information was available. Human airway fibroblasts (AFs) were isolated from bronchial tissue of transplanted COPD lung tissue using the explant method as described previously and approved for use in research [7].

The study protocol was according to the research code of the University Medical Center Groningen (https://umcgresearch.org/w/research-code-umcg accessed on 14 January 2025) and the national and ethical professional guidelines on the use of human body material (https://www.coreon.org/wp-content/uploads/2020/04/coreon-code-of-conduct-english.pdf accessed on 14 January 2025).

AECs were cultured in airway epithelium growth medium (AEGM; PromoCell, Heidelberg, Germany) supplemented with 10,000 units/mL penicillin/streptomycin (Gibco) in flasks coated with 3 mg/mL PureCol (Collagen; Advanced BioMatrix, Carlsbad, CA, USA), 1 mg/mL fibronectin bovine plasma (Sigma-Aldrich, Burlington, MA, USA), and 10 mg/mL BSA (Sigma-Aldrich) at 37 °C and 5% CO_2_.

AFs were cultured in Ham’s F12 (Gibco) supplemented with 10% FBS (Serana, Germany) + 10,000 units/mL penicillin/streptomycin (Gibco) at 37 °C and 5% CO_2_.

Cells were grown in 25 cm^2^ culture flasks and passaged with trypsin/EDTA once they were visually confirmed to be at ~90% confluency using light microscopy. The cells were seeded in the device at passage 3.

#### 2.4.2. Cell Seeding in the Device

AECs were seeded into the devices at a density of 6.5 × 10^4^ cells and AFs at a density of 8 × 10^4^ cells in 100 μL of culture medium and incubated overnight statically at 37 °C and 5% CO_2_ for cell attachment. Initially, the device was inverted, and fibroblasts were seeded overnight with inlets sealed before returning the device to its standard orientation when epithelial cells from 3 different donors were seeded into the apical chambers of parallel devices.

The cells were grown statically submerged with medium changing in both chambers every 24 h for 1–3 days. Upon reaching >90% confluency, AECs and AFs were imaged in brightfield on a Leica DM IL LED Inverted Laboratory Microscope with a Leica MC120 HD 2.5 Megapixel HD Microscope Camera (Leica microsystems, Wetzlar, Germany). Subsequently, the chips were connected to the Porcupine peristaltic pump.

#### 2.4.3. Cell Differentiation Under Airflow in the Millifluidic Device

AECs were grown to confluence and apically exposed to air in co-culture with AFs under continuous flow for 21 days at a constant rate of 150 μL/h. The circulating media were replaced with a full 1 mL reservoir of medium every 3 days in both instances. Cell-free supernatants and apical washes were collected at days 7, 14, and 21 and stored at −20 °C for up to two weeks before ELISAs were performed. For the secretion of mucus into the apical compartment, chips were incubated statically in 100 μL of respective media for 1 h before an apical wash was applied for 5 min and immediately stored at −20 °C for up to two weeks before performing the ELISA. The cells were washed with PBS, fixed in 4% PFA for 20 min, and whole chips were stored in PBS at 4 °C before staining.

#### 2.4.4. Immunofluorescent Staining

Primary cells were fixed onto the culture membrane with 1:1 ice cold methanol and acetone. Membrane segments were washed three times with PBS and incubated at room temperature for 1 h in 5% BSA for blocking and washed with PBS before incubation with primary antibodies.

For cell membrane staining, culture membranes were incubated with wheat germ agglutinin-conjugated AlexaFluor 555 (1:500, Abcam) and counterstained for 10 min with DAPI.

For immunohistochemical staining, the membranes were incubated for 1 h with the mouse anti-MUC5AC antibody (1:200, AbCam) and Rabbit-anti-FoxJ1 antibody (1:200, Lifespan Biosciences, Lynnwood, WA, USA), or Mouse-anti-α-SMA (1:200, AbCam) and Rabbit-anti-KRT-5-antibody (1:200, AbCam) in PBS containing 1% BSA. After washing with PBS, the cells were incubated for 1 h with AlexaFluor 555 donkey-anti-mouse and AlexaFluor 647 donkey-anti-rabbit-conjugated secondary antibody (1:200, Abcam) in PBS containing 1% BSA. Finally, the samples were incubated for 10 min with DAPI in PBS (1 µg/mL). The cells were washed in PBS before being fixed under CitiFluor (VWR). Images were taken with an SP8 Lightning Confocal Microscope using identical settings and pseudo-colored for the different targets (Leica Camera).

#### 2.4.5. Statistics

The data were analyzed using Student’s *t*-test for unpaired or paired observations, and one-way ANOVA was used to test for significance when time series were performed. Normal distribution of the data was assumed. *p* < 0.05 was considered statistically significant.

## 3. Results

### 3.1. Platform Validation

#### 3.1.1. The Large Scale Airway-on-Chip

**Figure 3:**
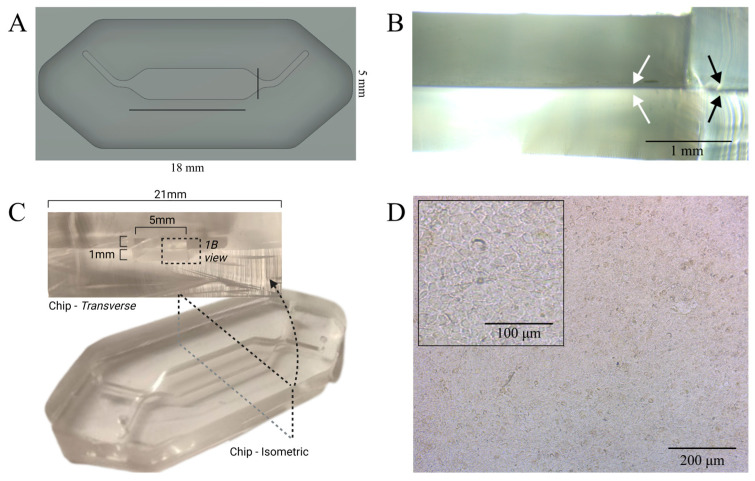
Airway-on-chip design and structure. (**A**) Outline of the airway-on-chip design, top-down image produced from a render of the mold within fusion360. (**B**) A transverse view down the length of the chip, the apical and basal chambers can be seen containing the white arrows, and the surrounding PDMS contains the black arrows. White arrows indicate the flat membrane placement resulting from the RT cure. Black arrows highlight the tight PDMS binding between the top and bottom halves around the PET membrane. (**C**) Transverse and isometric views of the actual device. This shows the outcome of the mold (**A**), the perspective seen magnified in (**B**), and the clear optical view through the culture chamber that was imaged through in (**D**). The inner PDMS surface of the apical chamber produced from the surface of the mold was micro-milled from the above design. The location of the zoom in the design is indicated by the white arrows. (**D**) Growth of epithelial airway cells within the airway-on-chip system. The images show clear optical resolution of representative cultures of a confluent monolayer of human epithelial lung cells (Calu-3) grown on the PET membrane (**B**) within the chip device.

Our chip was designed in such a way that each half of the chip consists of a body of PDMS, 5 mm deep for the top and 2.5 mm for the bottom, both with a single 1 mm-wide channel that opens into a 5 mm-wide cell culture chamber in the center (Figure 3A). The designs for the top and bottom are identical, except for the depth and two guide marks for punching inlets in the top half (Figure 3A). The inlets open into branches at 45° to the culture chamber to ensure separation of the two chamber’s flows across the membrane, with a short straight channel to precede and follow the culture chamber to prevent swirling of the incoming media (Figure 3C).

By cold- instead of hot-curing the PDMS mortar at room temperature under a vacuum for 72 h, we produced a large-scale airway-on-chip with a stable culture membrane (Figure 3B). Cold-curing prevented membrane displacement from occurring when the components cooled and shrunk at different rates. Applying the vacuum removed the toluene, any residual air bubbles, and promoted PDMS infiltration into the pores of the membrane, thereby ensuring a reliable water-tight seal (Figure 3B). A large culture area (92 mm^2^) was employed with the intention of producing a wide (5 mm) cell culture area with a stable media front under flow (Figure 3A). When Calu-3 cells were seeded into the chips, cell attachment and expansion occurred almost exclusively on the collagen-coated culture membrane, and not on the uncoated PDMS in the channels (Figure 3D).

#### 3.1.2. Cell Culture in the Millifluidic Device

For the quantification of the samples available from the device and comparison of ALI-induced differentiation between culture platforms, Calu-3 cells were grown in the different platforms to confluency. An example of a confluent cell monolayer in the microfluidic device can be seen in Figure 3D.

#### 3.1.3. Cell Viability in the Millifluidic Device

**Figure 4:**
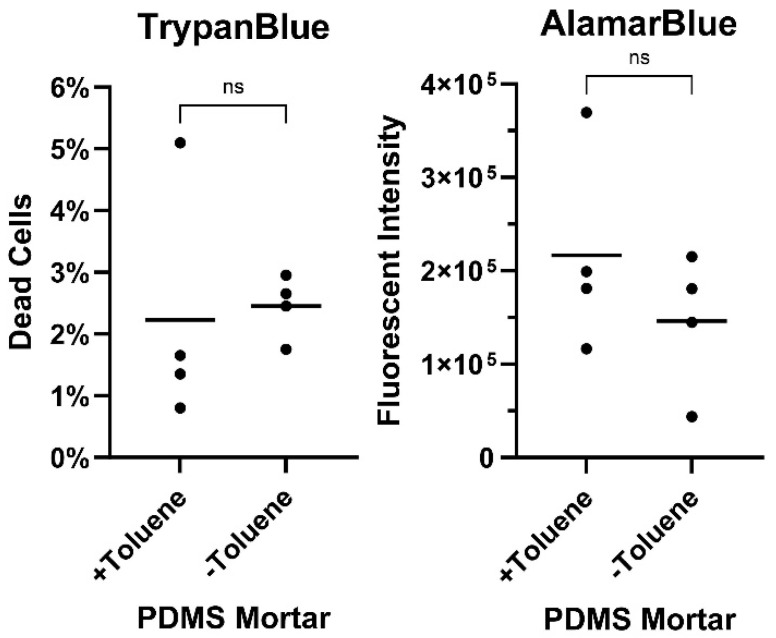
Cell viability in the chip devices was not altered by construction with or without toluene as a thinning agent in the PDMS mortar. Calu-3 cells were seeded at 6.5 × 10^4^ cells per chip and upon reaching confluency were incubated overnight before an AlamarBlue assay was performed on the supernatant and TrypanBlue on the cells. Differences between groups prepared with and without toluene were tested by unpaired Student’s *t* test, *p* > 0.5 = ns (not significant).

To investigate potential chemical toxicity from the use of toluene in the construction of the devices, the chips were prepared with and without toluene in a PDMS mortar, and cell death was assessed by Trypan blue and AlamarBlue^®^ cell viability assays (Figure 4).

We did not observe a significant difference in cell viability between cells grown in chips prepared with or without toluene. This indicates that the use of toluene as a thinning agent for the PDMS mortar was not toxic for the cells. This is of relevance, as the use of toluene in the mortar vastly improves membrane binding as well as the water-tightness of the final devices. As toluene is volatile and non-viscous, it acts to reduce the thickness of the liquid PDMS layer during construction [19].

#### 3.1.4. In-Chip Sample Quantification

To assess the capacity of the chips, we quantified the cell number, RNA yield, and protein content available from a confluent monolayer of Calu-3 cells grown inside the 92 mm^2^ culture chamber. To assess whether detectable levels of epithelial proinflammatory mediators were produced, IL-8 was measured in supernatants collected over 24 h (Table 1).

We observed that the cell numbers extracted from the devices were comparable to the quantities harvested from confluent Calu-3 cells in 24-well plates and to those grown exposed on Transwell inserts. The cell number present in the platform and the ability to extract samples from these cells indicate that the devices are suitable platforms for RNA isolation for qPCR or RNA-sequencing as well as protein analysis such as ELISA.

#### 3.1.5. Differentiation of Epithelial Cells Within the Airway-on-Chip

**Figure 5:**
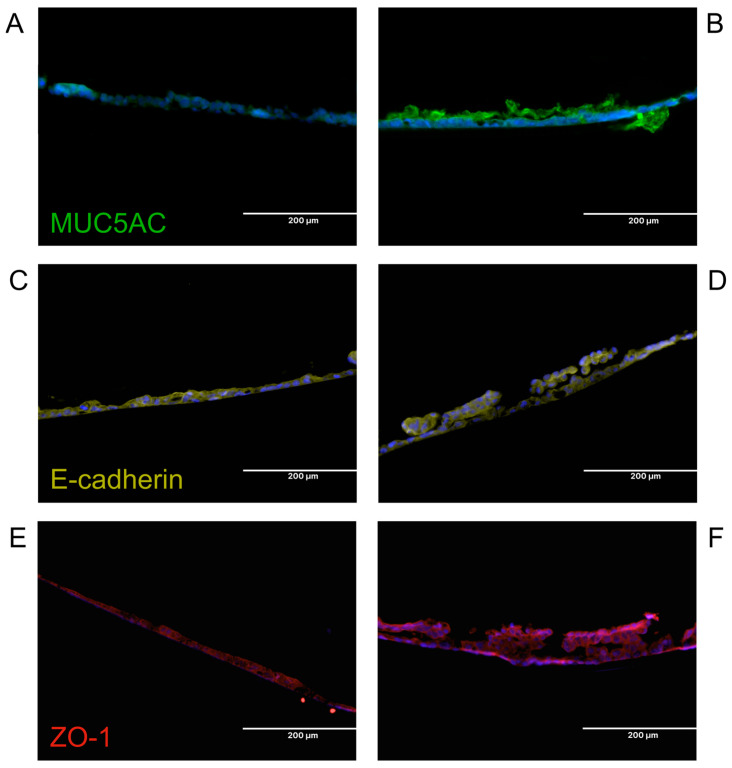
Junctional expression of cell–cell contact proteins in Calu-3 cells grown in the chips and mucus production after air exposure in the device. Calu-3 cells were grown to confluency and cultured for 10 days under a continuous flow rate of 150 μL/h in the basal compartment and either medium- or air-exposed from the apical side. After 10 days, the membranes were removed and stained. (**A**,**B**) Immunostaining of MUC5AC. (**C**,**D**) Immunostaining of E-cadherin. (**E**,**F**) Immunostaining of ZO-1. The left panels show stains performed on Calu-3 cells grown submerged for 10 days post confluency. The right panels show the stains on Calu-3 cells grown air-exposed for 10 days post confluency All fluorescent stains were counterstained with DAPI and pseudo-colored after imaging. Representatives images of 3 independent experiments are shown.

**Figure 6:**
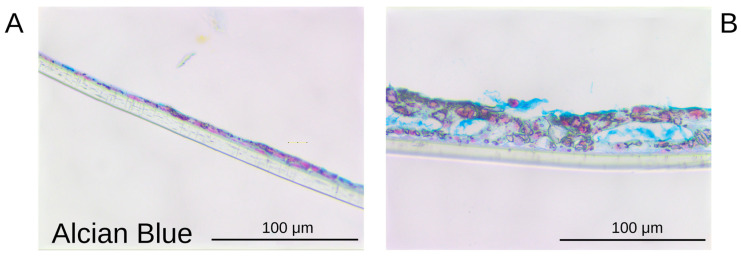
Alcian Blue staining of human lung Calu-3 epithelial cells grown in the chips after air exposure in the device. Calu-3 cells were grown to confluency and cultured for 10 days under a continuous flow rate of 150 μL/h in the basal compartment and either medium- or air-exposed from the apical side. At the end of culturing, the membranes were removed and stained. (**A**,**B**) Transverse view of Alcian Blue staining of Calu-3 cells grown for 10 days submerged (**A**) and air-exposed (**B**).

To validate the performance of the airway-on-chip as a platform for culture at the air–liquid interface, epithelial cells were air-exposed after establishing a confluent monolayer within the device. Upon 10 days of air exposure, Calu-3 cells demonstrated production of mucus (Figure 5A,B and Figure 6A,B). Upon day 21 of air exposure, primary epithelial cells and fibroblasts could be seen in the presence of mucus (Figure 5C,D). Furthermore, the Calu-3 cells displayed a more stratified layer upon air exposure with the presence of junctional localization of cell–cell contact molecules E-cadherin and ZO-1 throughout the layer (Figure 5C–F) as observed before in air-exposed Calu-3 cells [20,21]. In line with our findings, the formation of intracellular junctions upon air exposure has been reported previously in Transwell systems [31]. This indicates that our chip model facilitates the formation of epithelial junctions and the differentiation towards mucus-producing cells at the ALI.

**Figure 7:**
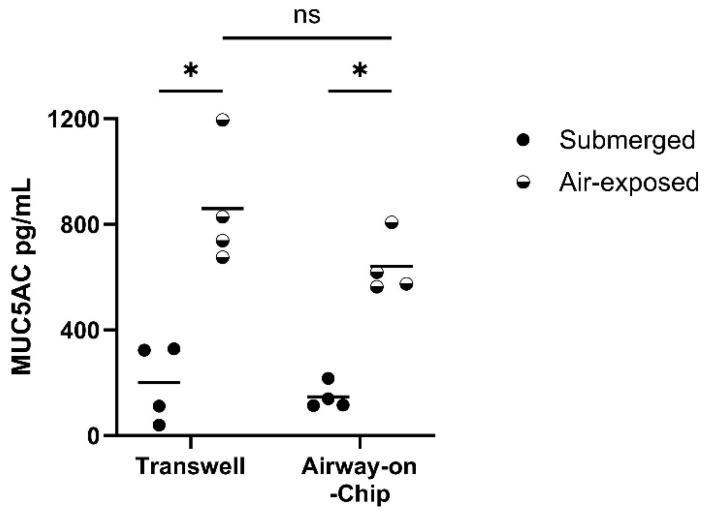
MUC5AC secretion from Calu-3 cells cultured in Transwell inserts and the airway-on-chip device was increased upon air exposure. Calu-3 cells were seeded in the chip device and on Transwell inserts and cultured until confluence, after which the cells were cultured submerged or air-exposed at 150 μL/h (medium and air) for 10 days, and apical washes were harvested to quantify MUC5AC secretion. Calu-3 cells (n = 4) grown for 10 days submerged or air-exposed on Transwell inserts (left) and in the airway-on-chip (right). * = *p* < 0.05, ns = *p* > 0.05 between indicated values as assessed by unpaired *t*-test.

In addition, we observed a significant increase in the secretion of MUC5AC in Calu-3 cells cultured under an airflow compared to that of submerged cultured Calu-3 cells in the chip device, similar to when cells were grown in well plates, without a significant difference between the culture platforms (Figure 7).

### 3.2. A Dynamic Primary Respiratory Cell Co-Culture ALI Platform

#### 3.2.1. Primary Airway Cell Co-Culture in the Airway-on-Chip

**Figure 8:**
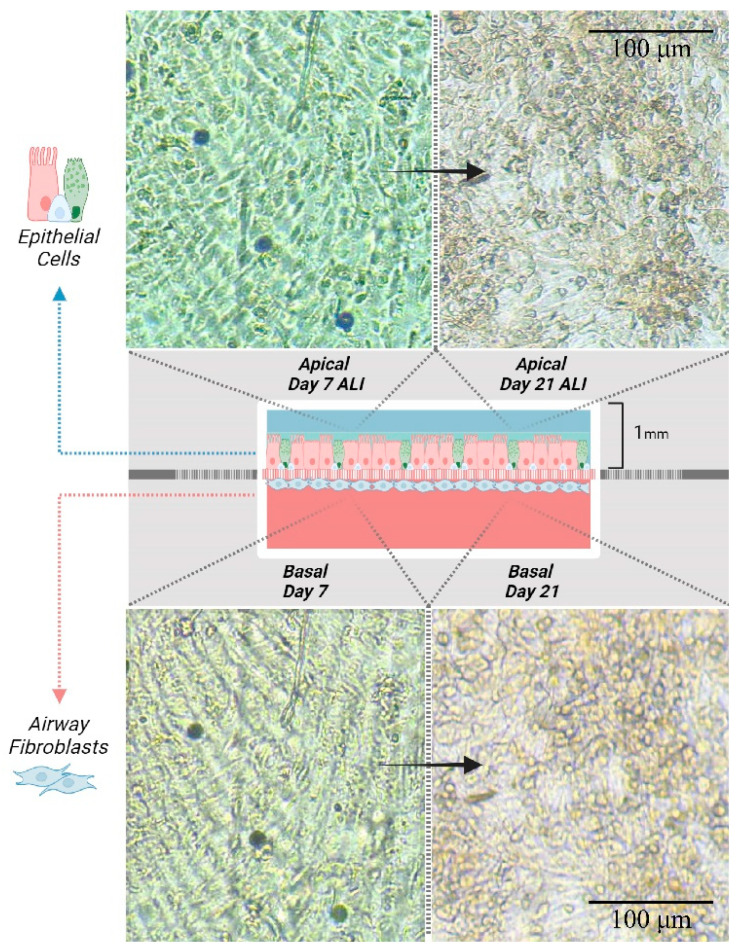
Growth of airway epithelial cells within the airway-on-chip system. Representative images of 3 independent cultures of human airway cells grown on the PET membrane within the chip device are shown. Airway fibroblasts were cultured on the basal side of the membrane, with epithelial cells cultured on top. After the cells reached confluency, the cultures were air-exposed from the apical compartment. The cells were images on days 7 and 21, and images of both layers of cells were taken top-down in the same position. Epithelial cells in the apical chamber can be seen above, fibroblasts on the other side of the membrane in the basal chamber can be seen below both at day 7 upon air exposure on the left and at day 21 on the right.

**Figure 9:**
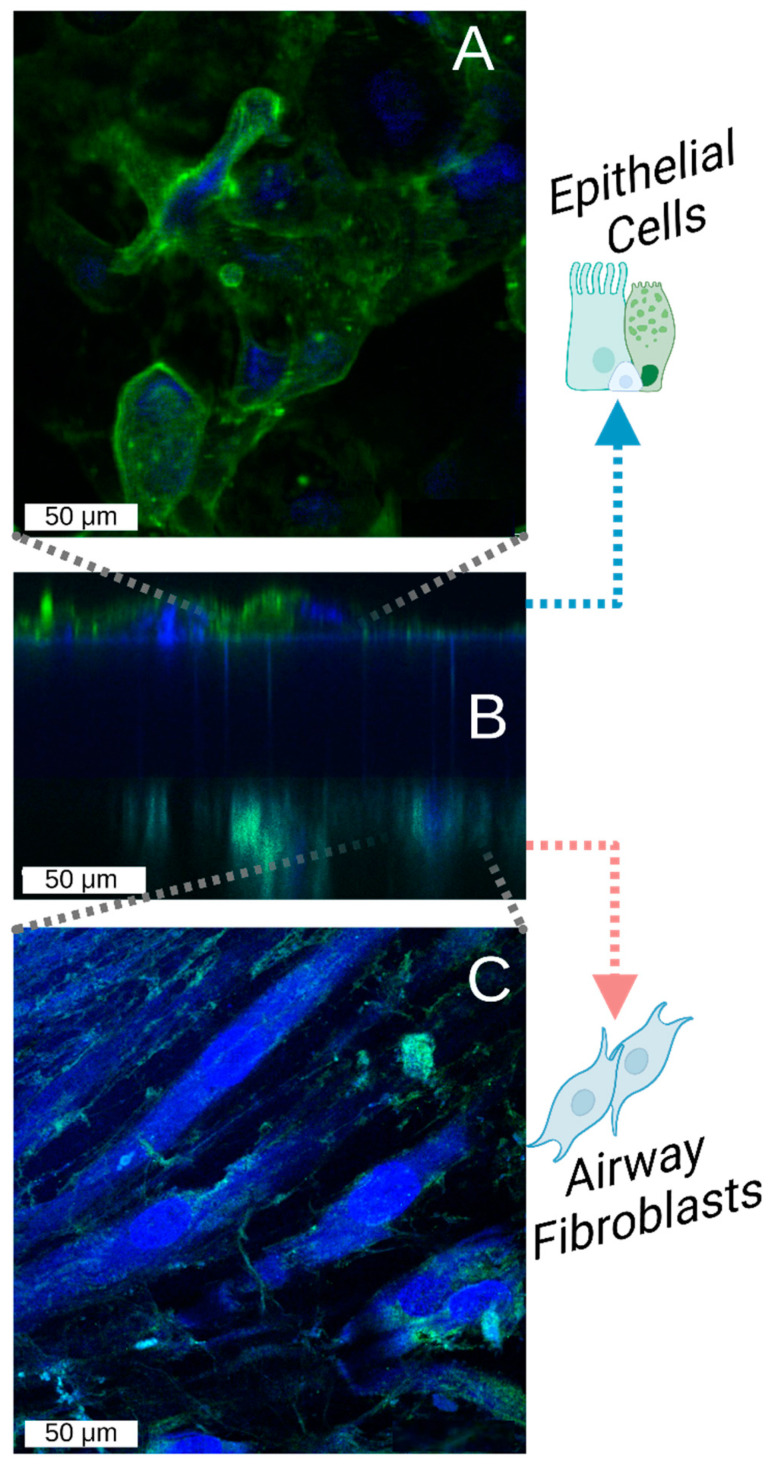
Identification of epithelial cells and fibroblasts grown within the device. Airway fibroblasts were seeded into the device and allowed to attach overnight before inverting the device and seeding epithelial cells on the other side of the membrane. The cell were grown to confluency, air-exposed, and cultured for 21 days under a continuous flow rate of 150 μL/h. The membranes were removed post-culture, fixed, embedded, and mounted on slides before staining. All cells were stained with wheat germ agglutinin (WGA) to visualize the cellular phospholipid bilayers and their counterstained with DAPI. (**A**) Complete external structure of the epithelial layer in the apical chamber (transverse). (**B**) Cross-section view: produced from a Z-stack projection of images underlying (**A**,**C**) to visualize the location of the cells around the culture membrane within the device. (**C**) Complete external structure of the fibroblast layer in the basal chamber (transverse).

**Figure 10:**
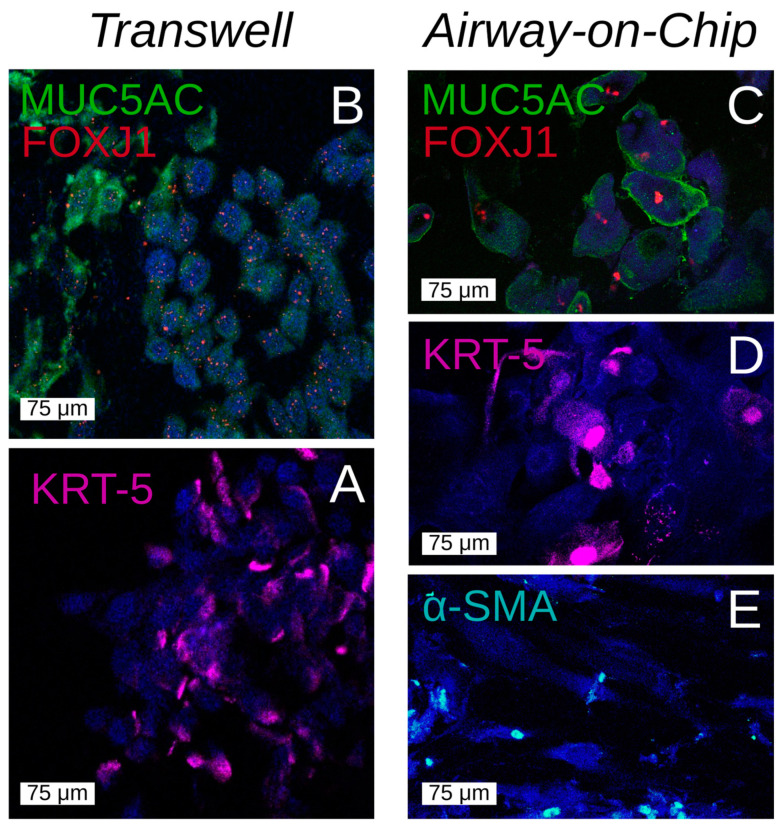
Differentiation markers in airway epithelial cells cultured at the air–liquid interface (ALI) in static Transwells and co-cultured airway epithelial cells and fibroblasts in the airway-on-chip model. Airway epithelial cells were seeded into Transwell inserts or chips with fibroblasts cultured on the other side of the membrane. Once the cells reached confluency, they were exposed to air for 21 days and fixed for confocal staining. The cells were under a continuous flow rate of 150 μL/h in both the apical and basal compartment (air or medium). The top panels show stains for Mucin 5AC (MUC5AC), a component of mucus, forkhead box protein J1 (FOXJ1), a transcription factor involved in signaling for cilia production. The panels below show stains for cytokeratin-5 (KRT-5), a basal epithelial cells marker, and the bottom right panel shows alpha-smooth muscle actin (α-SMA) a cytoskeletal element that is specific to fibroblasts. All fluorescent stains were counterstained with DAPI and pseudo-colored after imaging. (**A**) MUC5AC and FOXJ1 on an ALI insert, (**B**) keratin (KRT)-5 stain on an ALI insert, (**C**) MUC5AC/FOXJ1 stain on a chip membrane. (**D**) KRT-5 stain on a chip membrane, (**E**) α-smooth muscle actin (SMA) stain of fibroblasts in the basal chamber of the same chip membrane as above.

To validate our model for prolonged epithelial–fibroblast co-culture, primary airway epithelial cells were cultured under airflow exposure for 21 days in close proximity to airway fibroblasts, which were cultured upside down on the other side of the membrane. In Figure 8, a graphical representation of the chip as it was imaged can be seen. The co-culture was imaged at the beginning (day 7) and end (day 21) of the culture period. Above the images taken of the epithelial cells in the apical chamber, below the graphic, aligned images of the fibroblasts in the basal chamber (Figure 8). The presence of fibroblasts on the basal side of the membrane and epithelial cells on the apical side of the membrane was confirmed by wheat germ agglutinin staining, which stains all cell membranes with GFP, showing the different morphology of the more rounded epithelial cells in the apical chamber (Figure 9A) and the elongated mesenchymal cells in the basal chamber (Figure 9C). Whole co-cultured membranes were imaged by confocal microscopy into a z-stack and digitally reconstructed into a side-view with imageJ (Z-stack project; Figure 9B). Furthermore, to identify the differentiation state of the cells, we performed immunohistochemical staining on the different type of epithelial cells present on the apical side of the membrane, similar to epithelial cells cultured under conventional ALI conditions (Figure 10A,B), including basal cells expressing cytokeratin (KRT) [5], goblet cells producing MUC5AC, and cells expressing ciliated cell-specific transcription factor FOXJ1 (Figure 10C,D). These markers have previously been shown to be expressed in differentiated airway epithelial cells upon air exposure, but not in undifferentiated, submerged cultured cells [31]. Of note, the overall decrease in cell size observed upon static ALI culture was not observed in the microfluidic device. Additionally, the fibroblasts grown on the basal side were positive for mesenchymal cell marker α-smooth muscle actin (SMA, Figure 10E).

#### 3.2.2. Differentiation of Epithelial Cells Within the Airway-on-Chip

**Figure 11:**
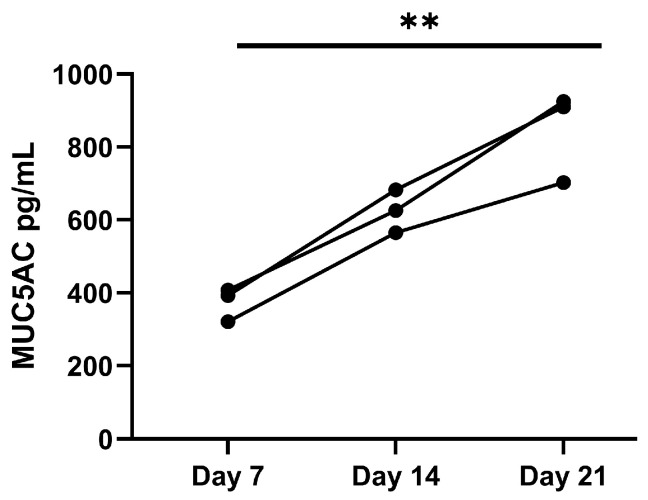
MUC5AC secretion in human airway epithelial cells (AECs) cultured in the airway-on-chip device was increased upon air exposure. AECs (n = 3) were seeded in the chip devices and cultured until confluence, after which the cells were air-exposed at 150 μL/h air for 7–21 days, and apical washes were harvested to quantify MUC5AC secretion. ** = *p* < 0.01 between the indicated values as assessed by one-way ANOVA.

To further validate the performance of the airway-on-chip as a platform for epithelial differentiation at the air–liquid interface, the levels of MUC5AC were quantified in the in the apical fluid collected during differentiation from days 7 and 21 of air exposure. MUC5AC levels significantly increased from day 7 to 21 (Figure 11), indicating that our chip model not only facilitated the long-term co-culture of AECs and AFs, but also allowed for the differentiation of the epithelial layer towards mucus-producing goblet cells.

#### 3.2.3. Fluid Dynamic Analysis Within the 3D Geometry of the Device

**Figure 12:**
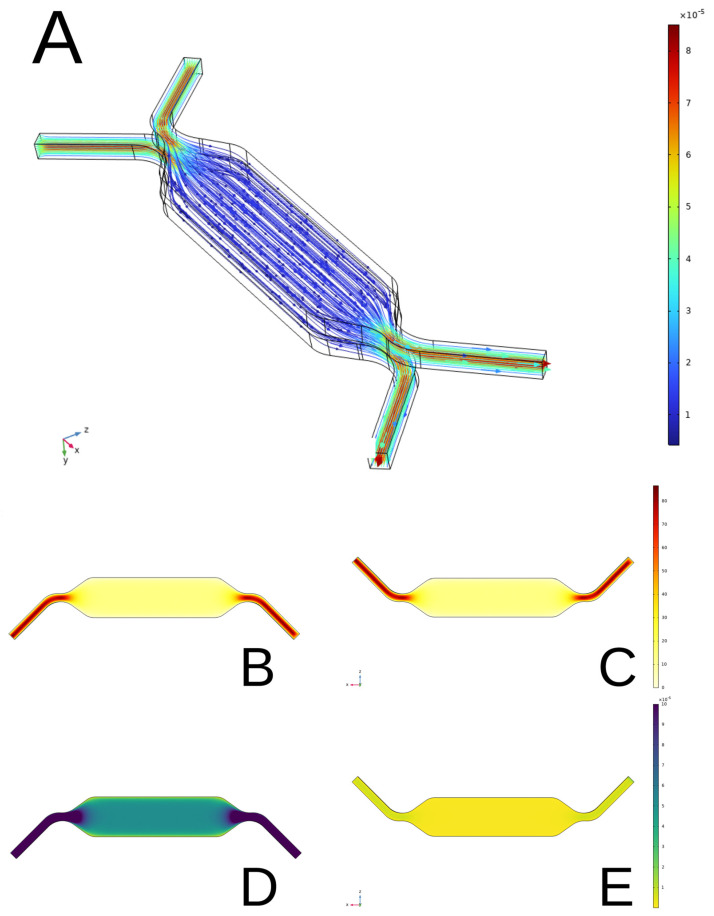
Simulations of media and air movement through the geometry of the device. (**A**) Streamline simulations of air/media flow showing laminar flow directionality throughout the device (m/s). (**B**) Media velocities (μm/s) in the basal media chamber. (**C**) Media velocities (μm/s) in the apical air chamber. (**D**) Shear stress (Pa) exerted by media on the wall of the basal chamber. (**E**) Shear stress (Pa) exerted by air on the wall of the apical chamber.

To visualize the laminar directionality and distribution of media within the device, the fluid and airflow simulation results were evaluated as follows in post-processing. This phase allows a visual display of the device to be obtained to evaluate the results. The visualization was performed using plots or different types of graphical displays. The type of plots used were slice plots, streamline plots, and surface plots. Finally, to visualize the data, a streamline plot and graphs were produced to highlight the maximum value assumed by the variables (media velocity/shear stress) within the microfluidic channels (Figure 12).

## 4. Discussion

The validation of our newly developed airway-on-chip model showed that the device can be utilized to study differentiated, air-exposed respiratory epithelial cells under a biological flow, allowing for the assessment of effects of airborne exposures and volatile factors released into the airflow. The addition of directional flow in our chip provides a simulation of the biological shear stress found naturally in the airways as well as facilitates the sampling of secreted factors in a controlled, non-invasive manner.

While more specialized microfluidics with open and available construction methods are available in the literature that feature more fine-tuned control of shear stresses [12], and more advanced dynamic ALI platforms such as the Emulate chip are more consistent due to their mass production, our device provides access to such a platform without the complex 3D construction or the requirement for electrospinning to produce the stretchable membrane [11,18]. This allows our device to be constructed and applied to a range of lab environments and experimental setups without the large investment into setting up and running commercial platforms. Additionally, as the input materials for this device are cost-effective, it opens avenues for more exploratory and high-throughput airway-on-chip projects.

While the device has an achievable construction procedure and functions as a dynamic ALI platform, it also has some limitations. Firstly, although the entire construction procedure is possible with a resin 3D printer, the surface distortion on the resultant PDMS from their screen resolutions appeared to limit imaging possibilities. To solve this, production of the molds needs to be outsourced to a facility with a micro-mill that compromises the ease of design and construction but creates permanent molds as a one-time investment over printed molds, which expire over time.

Furthermore, as there are no stretchable PDMS membranes or standardized ECM membranes for cell cultures on the market, basic PET culture membranes have been implemented. However, the implementation of a cyclical stretch would require only the addition of a PDMS membrane by O_2_ plasma bonding or ECM by our RT-cure PDMS Mortar method, which could be implemented in the device if the facilities were available. Preliminary tests with basement membranes extracted from pig bladders have shown that the same air-tight seal is achievable through the RT PDMS Mortar curing methods we describe here.

In the future, this device is intended for use with a range of particulate exposures, from whole cigarette to e-cigarette vapor. Furthermore, with the possibility of culturing high cell numbers within the device, the model is highly suited for multi-organ studies, potentially looking into the systemic effect of respiratory exposures on other organ compartments. The design may also facilitate the detection of (volatile) metabolites because of the potential for primary differentiation and the high ratio of cells to headspace within the device.

## 5. Conclusions

We show that this airway-on-chip has an accessible production methodology, substantial sample capacity, and facilitates the differentiation of human epithelial airway cells under air exposure. With directional airflow in the wide apical chamber, the device provides a platform to study respiratory epithelial differentiation and its dysregulation in disease at the scale of the conducting bronchi (≤5 mm). The flow through the cell culture area allows for near-continuous sampling of the factors released by the epithelial layer. By accessing these flows via reservoirs, sampling and stimulations can be achieved in either air or liquid media, without any direct interactions with the culture. Our device is capable of dynamic culturing over extended periods and facilitates the co-culture of other cells types in its basal chamber alongside a differentiated epithelium. This airway-on-chip device may be constructed with minimal investment in materials and equipment, providing a cost-effective alternative to commercial systems. Together, this airway-on-chip constitutes a highly accessible improvement on static ALI and small-scale dynamic culture platforms.

## Figures and Tables

**Table 1:** Quantification of samples from a confluent layer of Calu-3 cells cultured in multiple culture platforms. The cells in the chip device were air-exposed for 10 days, the cells in well plates were cultured submerged for 10 days, and the cells in the Transwell system were air-exposed for 10 days.

Sample Type	Total Quantity	Quantity per Unit
**Cell number**		
*Chip*	162,500 ± 30k	1800 cells/mm^2^
*24-well*	225,000 ± 35k	1200 cell/mm^2^
*Transwell*	62,000 ± 8k	1900 cells/mm^2^
**RNA**		
*Chip*	1300 ± 300 ng	10–22 ng/mm^2^
*24-well*	1900 ± 400 ng	14 ng/mm^2^
*Transwell*	480 ± 100 ng	15 ng/mm^2^
**Total protein**		
*Chip*	237.5 ± 20 μg	2600 μg/mL
*24-well*	420.2 ± 40 μg	2200 μg/mL
*Transwell*	95.5 ± 15 μg	2900 μg/mL
**IL-8**		
*Chip*	49 ± 12 pg	530 pg/mL

Values are the mean ± standard deviation of *n* = 4 independent experiments.

## Data Availability

The raw data supporting the conclusions of this article will be made available by the authors on request. Stereolithography (stl) files for 3D printing of the top and bottom parts of the airway-on-chips as well as the membrane stencil mold have been included as part of the Supplementary Information.

## Supplementary Materials

The following supporting information can be downloaded at: https://www.mdpi.com/article/10.3390/bioengineering12020182/s1, Supplement File: top_mould.stl/.png, bottom_mould.stl/.png, membrane_stencil.stl/.png.

## Abbreviations

The following abbreviations are used in this manuscript:

PDMS	polydimethylsiloxane
PET	polyethylene
ALI	air–liquid interface
ECM	extracellular matrix
ZO-1	zonula occludens 1
COPD	chronic obstructive pulmonary disease
.STL	standard tessellation language
PMMA	polymethyl methacrylate
EMEM	minimum essential Eagle’s medium
BSA	bovine serum albumin
DMEM	Dulbecco’s modified Eagle’s medium
FBS	fetal bovine serum
HBSS	Hanks’ balanced salt solution
PBS	phosphate-buffered saline
PFA	paraformaldehyde
AECs	airway epithelial cells
AFs	airway fibroblasts
AEGM	AEGM
KRT-5	cytokeratin-5
SMA	smooth muscle actin
WGA	wheat germ agglutin
MUC5AC	Mucin 5AC
FOXJ1	forkhead box protein J1
RT	room temperature
