# Supplementary figures and images for: Development of a Widely Accessible, Advanced Large-Scale Microfluidic Airway-on-Chip
Source: Bioengineering (Basel). 2025 Feb 13;12(2):182. doi: 10.3390/bioengineering12020182 (PMC11851814; doi:10.3390/bioengineering12020182)

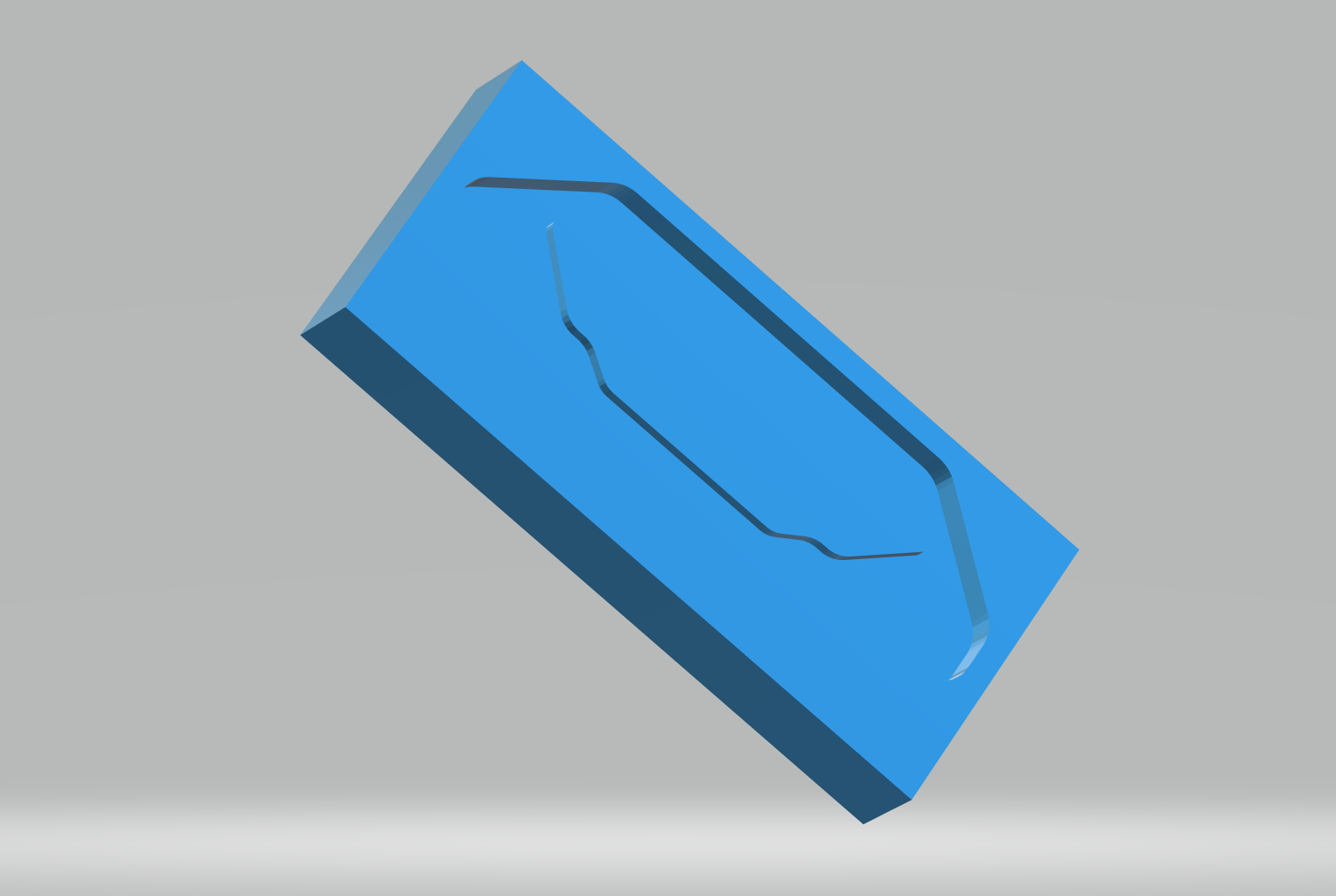

Supplement: Supplementary file 1 [file bioengineering-12-00182-s001.zip › bioengineering-3417138-supplementary/Bottom Mold.png]

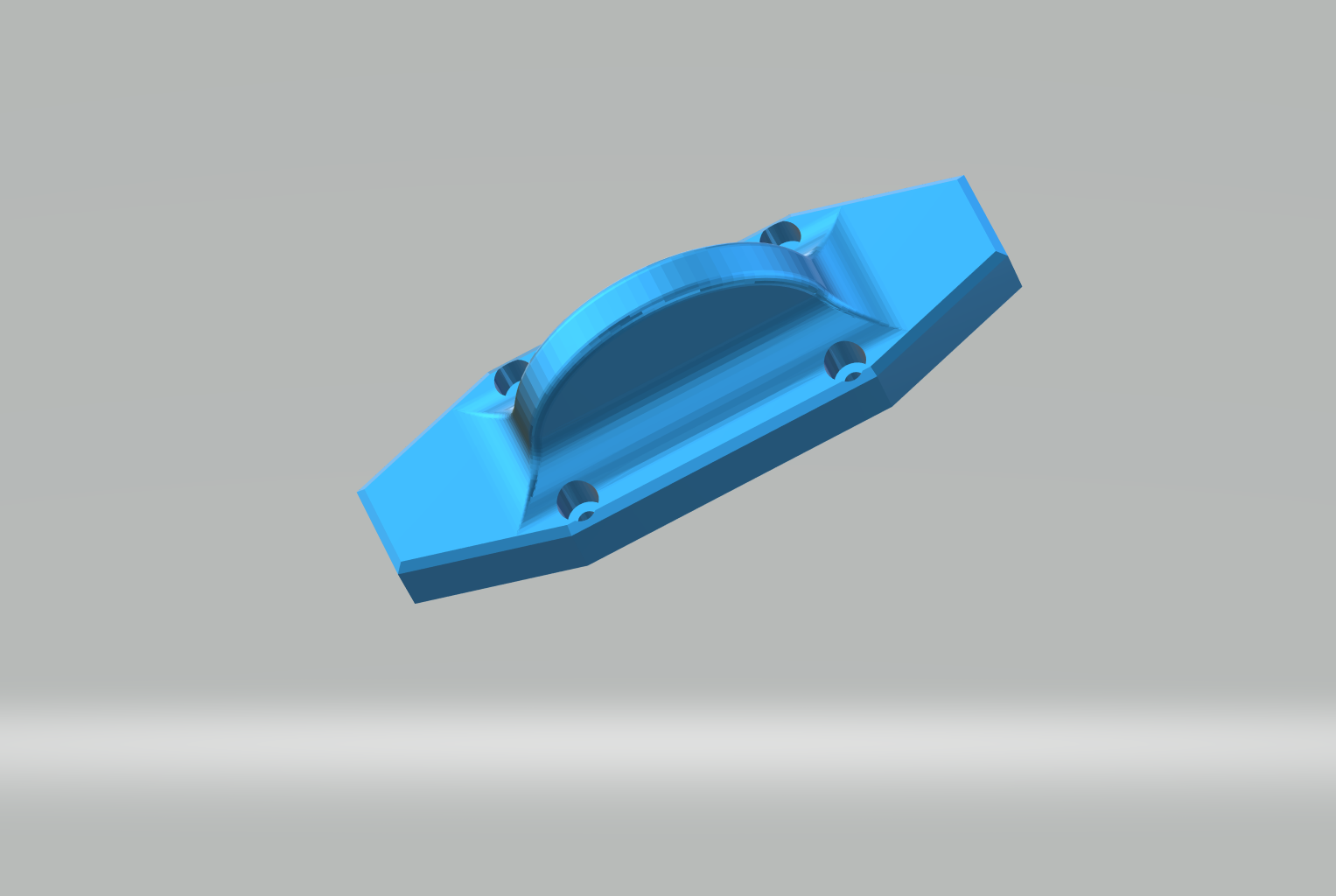

Supplement: Supplementary file 1 [file bioengineering-12-00182-s001.zip › bioengineering-3417138-supplementary/Membrane Stencil 2.0.png]

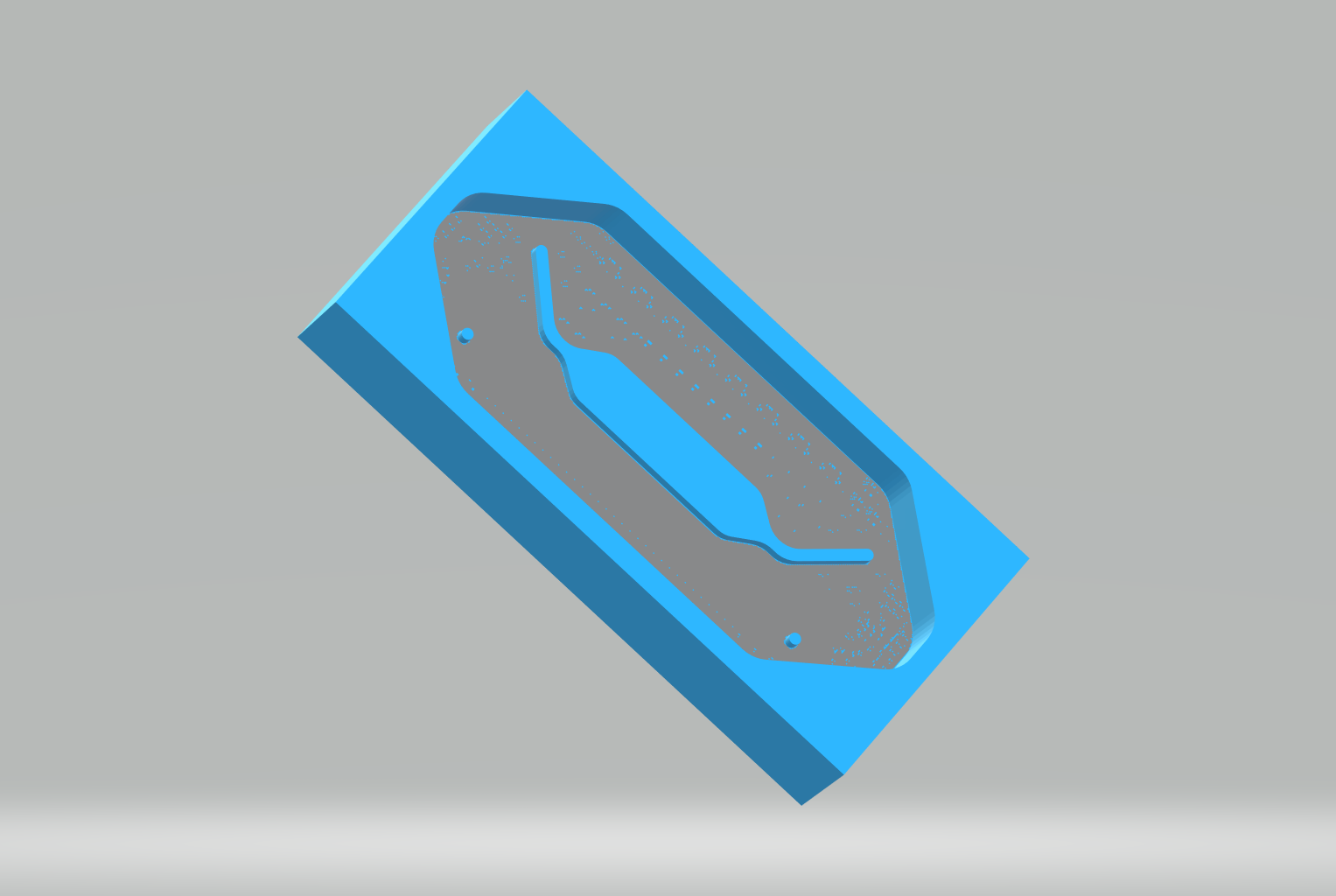

Supplement: Supplementary file 1 [file bioengineering-12-00182-s001.zip › bioengineering-3417138-supplementary/Top Mold.png]
